# Excellent functional outcomes without physeal violation after arthroscopic all‐inside ligament repair for ankle instability in skeletally immature patients

**DOI:** 10.1002/jeo2.70683

**Published:** 2026-03-11

**Authors:** Matteo Guelfi, Jorge Knörr, Giovanni Ruscigno, Francesc Malagelada, Andrea Pantalone, Jordi Vega

**Affiliations:** ^1^ Foot and Ankle Unit Casa di Cura Villa Montallegro Genoa Italy; ^2^ Clinica Salus, Gruppo Policlinico di Monza Alessandria Italy; ^3^ J Medical Turin Italy; ^4^ MIFAS by GRECMIP Merignac France; ^5^ Pediatric Orthopedics Hospital de la Santa Creu i Sant Pau Barcelona Spain; ^6^ Department of Orthopedic Surgery Azienda Ospedaliero‐Universitaria Careggi, University of Florence Florence Italy; ^7^ Foot and Ankle Unit, The Royal London Hospital Barts Health NHS Trust London UK; ^8^ Department of Medicine and Science of Aging University G. d'Annunzio, Chieti‐Pescara Chieti Italy; ^9^ iMove Sports Barcelona Spain

**Keywords:** ankle arthroscopy, children, chronic ankle instability, ligament repair, skeletally immature

## Abstract

**Purpose:**

Arthroscopic all‐inside ligament repair is a widely used technique for treating chronic lateral ankle instability. This study aimed to evaluate the clinical outcomes of skeletally immature patients who underwent all‐inside ligament repair.

**Methods:**

Skeletally immature patients who underwent surgery for ankle instability between 2018 and 2022 were retrospectively evaluated. All patients were treated arthroscopically using an all‐inside ligament repair technique, allowing an all‐epiphyseal anatomical repair of the ligament remnant. Clinical outcomes were assessed pre‐ and postoperatively using Visual Analogue Scale (VAS), ﻿Foot Functional Index (FFI) score and Foot and Ankle Ability Measure–Sports subscale (FAAM‐SS). Postoperative magnetic resonance imaging (MRI) was routinely performed at 3 months to evaluate ligament repair and detect any physeal violation. At final follow‐up, patient satisfaction, complications, lower‐limb axial deviations or clinical signs of physeal violation were recorded.

**Results:**

Forty patients aged 8–15 years (21 females; mean age 13.8 ± 2.3 years) were included. Mean follow‐up was 44.8 months ± 16.5 (range, 24–78). An anterior talofibular ligament (ATFL) tear was identified in all cases, with concomitant calcaneofibular ligament detachment observed in four patients. All clinical scores improved significantly (*p* < 0.001). Mean FFI and FAAM‐SS scores improved from 35.6 ± 14.0 (range, 11–69) and 50.3 ± 14.0 (range, 15–78) preoperatively to 3.9 ± 6.4 (range, 0–26) and 95.4 ± 8.3 (range, 68–100) postoperatively. Mean VAS improved from 5.4 ± 2.5 (range, 1–8) to 0.4 ± 0.8 (range, 0–3). No cases of physeal violation were detected on postoperative MRI. No major complications, axial deviation or clinical signs of growth plate injury were observed.

**Conclusions:**

Ankle instability in skeletally immature patients can be successfully treated with arthroscopic all‐inside ligament repair. This technique provides an all‐epiphyseal anatomical repair of the ligament remnant with a low risk of physeal violation and results in excellent clinical outcomes.

**Level of Evidence:**

Level IV, retrospective case series.

## Graphical abstract

Arthroscopic all‐inside ligament repair in skeletally immature patients yields excellent functional outcomes without physeal violation for ankle instability.

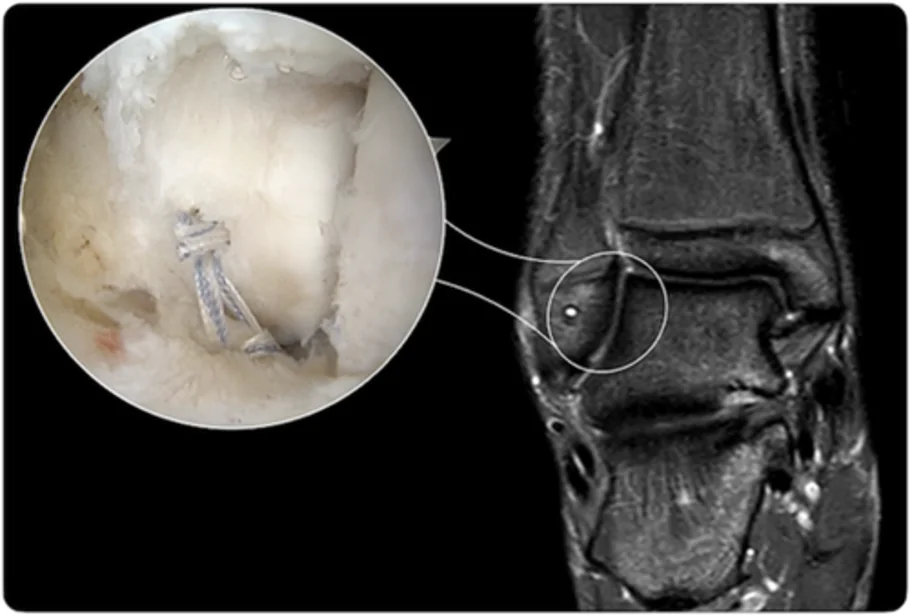

**Abbreviations**
ATFL: anterior talofibular ligament
CAI: chronic ankle instability
CFL: calcaneofibular ligament
FAAM‐SS: Foot and Ankle Ability Measure—Sports Subscale
FFI: Foot Functional Index
IRB: institutional review board
MRI: magnetic resonance imaging
ROM: range of motion
VAS: Visual Analogue Scale

## INTRODUCTION

Ankle sprains are one of the most common orthopaedic injuries, with the lateral ligament complex being involved in up to 85% of cases [4, 8]. In adults, approximately 20%–40% of ankle sprains involving the lateral ligaments ﻿may evolve to chronic lateral ankle instability ﻿and require a surgical treatment [5, 8]. The incidence appears to be similar—or even higher—in children and adolescents [12, 17, 20].

Traditionally, ankle instability in skeletally immature patients has been managed conservatively with bracing and physiotherapy [9, 12]. However, if left untreated or inadequately addressed, persistent instability can lead to early‐onset osteoarthritis and various intra‐articular pathologies, including soft tissue and bony impingements, osteochondral lesions and loose bodies—particularly when instability develops at a young age [6]. For this reason, surgical stabilization is recommended in cases of persistent instability or when daily activities and sports performance are significantly impaired [7, 9].

The increasing involvement of children and adolescents in sports has led to a corresponding rise in ankle instability within this population. Consequently, the number of surgical stabilizations performed in paediatric and adolescent patients has increased over recent decades.

In the adult population, arthroscopic all‐inside ligament repair has recently demonstrated similar or superior clinical outcomes when compared to the open repair [10, 11, 27]. For this reason, it has gained widespread popularity and has become the preferred method for many surgeons [21, 22]. However, in skeletally immature patients, few studies have evaluated the clinical outcomes of arthroscopic ligament repair, and the existing literature remains limited. Theoretically, the all‐inside technique allows for anatomic reattachment of the ligament remnant to its native footprint without violating the growth plate, making it a potentially suitable option for skeletally immature patients.

The aim of this retrospective study was to evaluate the clinical outcomes of arthroscopic all‐inside ligament repair in skeletally immature patients. A case series of 40 patients was analysed. To date, no other studies have investigated the results of all‐inside ligament repair in addressing ankle instability in this population.

The hypothesis was that arthroscopic all‐inside ligament repair could be an effective treatment for ankle instability and that it would yield excellent clinical results in skeletally immature patients.

## MATERIALS AND METHODS

﻿The study protocol was conducted in accordance with the principles of the Declaration of Helsinki and received ethical approval from the institutional review board of Clinica Salus, Alessandria (Italy) (IRB number 2021‐09‐020).

This retrospective study reports descriptive data presented as mean ﻿± standard deviation. All skeletally immature patients who underwent arthroscopic treatment for chronic ankle instability (CAI) between 2018 and 2022 (a 5‐year period) were considered for inclusion. Inclusion criteria included skeletally immature patients with open physis on preoperative x‐rays and a history of ankle instability lasting at least 6 months. Before surgery, a minimum of 3 months of protocolized physiotherapy ﻿(anti‐inflammatory therapy, rest and physiotherapy) was completed without symptom improvement. Conservative treatment was considered failed in cases of persistent lateral ankle pain or discomfort, recurrent ankle sprains, subjective feeling of ankle instability and limitations in daily activities.

Patients whose parents or legal guardians did not agree to participate in the study and refused to sign informed consent were excluded. Patients with previous foot and ankle surgery, lower‐limb malalignment or the presence of talar osteochondral lesions or peroneal tendon pathology were also excluded. ﻿Cases of generalized ligamentous laxity (Beighton score ≥ 6) and neuromuscular diseases were also discarded from the study. The observation of a calcaneofibular ligament (CFL) tear in addition to the anterior talofibular ligament (ATFL) tear in the preoperative magnetic resonance imaging (MRI) was not an exclusion criterion.

The diagnosis of CAI was established based on clinical history, standardized physical examination and 1.5 T MRI findings, all assessed by a single orthopaedic surgeon with expertise in foot and ankle surgery. The clinical history included recurrent ankle sprains or a sensation of giving way for a minimum of 6 months. Clinical examination focused on identifying anterolateral pain, a positive anterior drawer test and a varus talar tilt test compared with the contralateral side.

﻿Standard weight‐bearing radiographs were obtained in all cases to confirm the skeletal maturity and assess the status of the physis. A standard 1.5 T ankle MRI was obtained preoperatively in all cases and postoperatively at 3 months to assess ligament repair and anchor proximity to the physis or any physeal violation.

Functional outcomes were assessed preoperatively and at the latest follow‐up (minimum 1 year postoperatively) using the Foot Function Index (FFI), Visual Analogue Scale (VAS) and Foot and Ankle Ability Measure–Sports Subscale (FAAM‐SS). ﻿Preoperative and latest follow‐up ankle anterior drawer test scores and range of motion (ROM) were compared with the healthy contralateral side and recorded. At the final follow‐up, patient satisfaction, any complications, lower‐limb axial deviations or clinical signs of physeal violation—such as localized pain, swelling and tenderness directly over the growth plate—were recorded.

### Surgical technique

﻿All procedures were performed by two independent foot and ankle surgeons. Each surgeon performed an equal number of procedures and followed the same standardized surgical technique.

A no‐distraction dorsiflexion arthroscopic technique was used. Patients were positioned supine under spinal anaesthesia with a thigh tourniquet. Arthroscopic equipment included a 4.0‐mm 30° arthroscope, a 3.5‐mm motorized shaver and standard arthroscopic instruments.

Standard anteromedial and anterolateral portals and an accessory lateral portal were created. During arthroscopic examination, any intra‐articular pathology—including synovitis, fibrous tissue or loose bodies—was addressed as necessary. The lateral collateral ligament was then examined and repaired according to the original technique as described for the skeletally mature patients [23, 24].

Specific instruments for ligament repair included a curved 70° suture passer (﻿Microsuture lasso; Arthrex), nonabsorbable #0 sutures (Fiberwire; Arthrex) and knotless suture anchors (Pushlock 2.9 mm × 12.5 mm; Arthrex).

**Figure 1:**
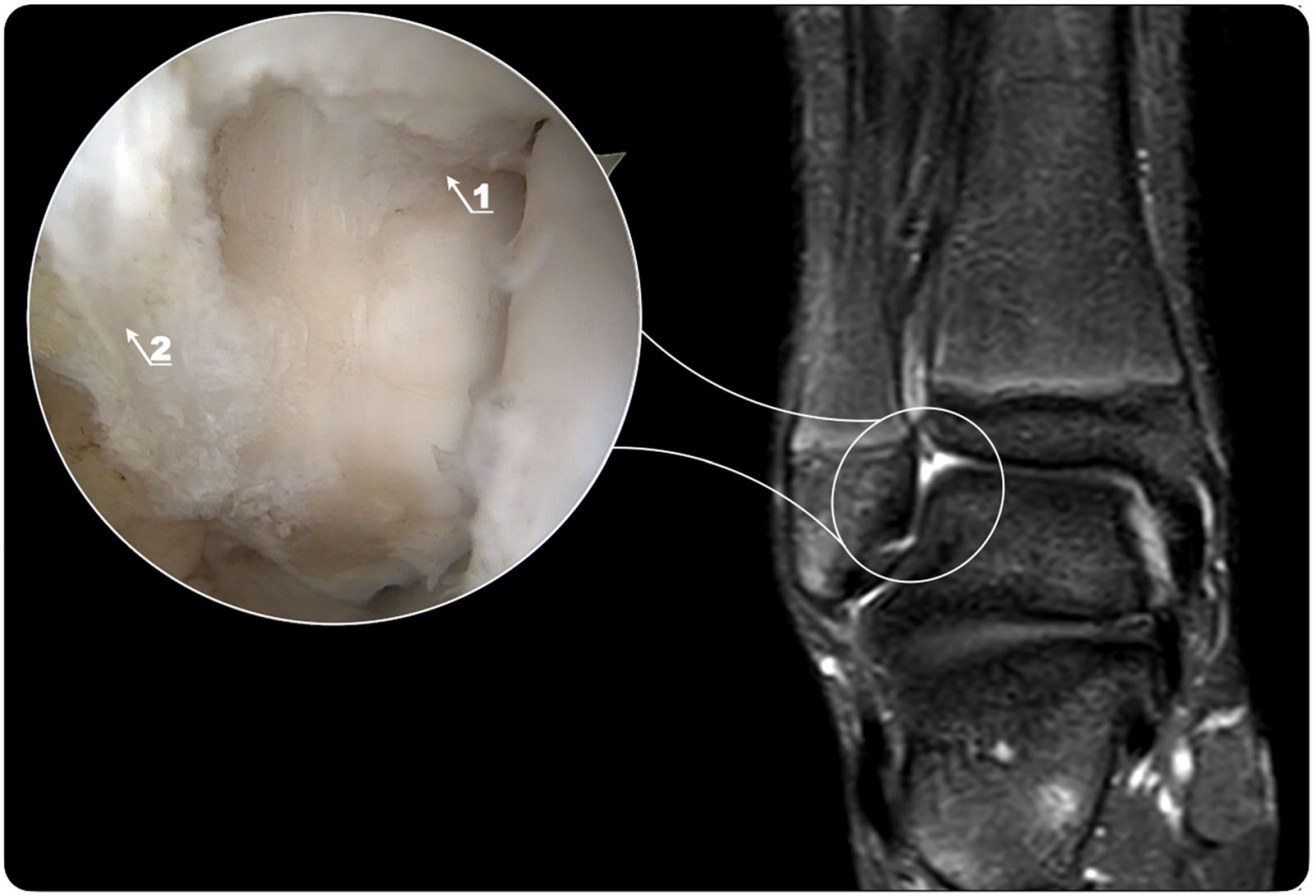
Preoperative MRI (right) and corresponding arthroscopic view of the lateral gutter (left) showing the fibular physis (1) at the same level as the joint line and located behind the anterior inferior tibiofibular ligament (2). MRI, magnetic resonance imaging.

**Figure 2:**
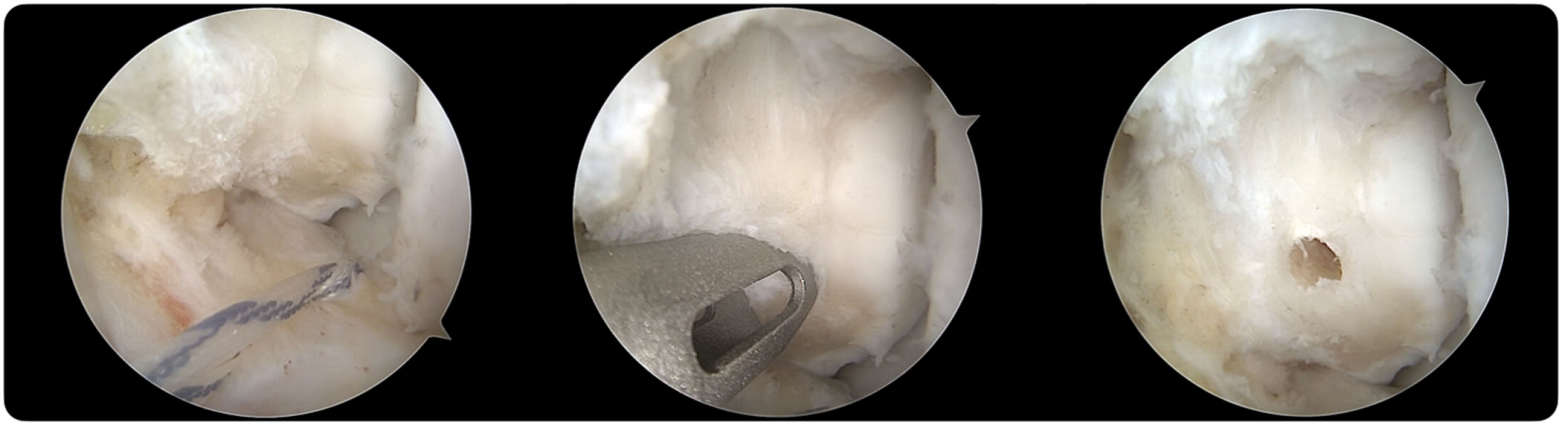
Step‐by‐step all‐inside repair of the ATFL. The ligament remnant is grasped with a high‐resistance suture. The drill guide is then located just distal to the fibular attachment of the anterior inferior tibiofibular ligament, and the distance between the fibular physis and the intended anchor location is carefully checked before drilling. ATFL, anterior talofibular ligament.

**Figure 3:**
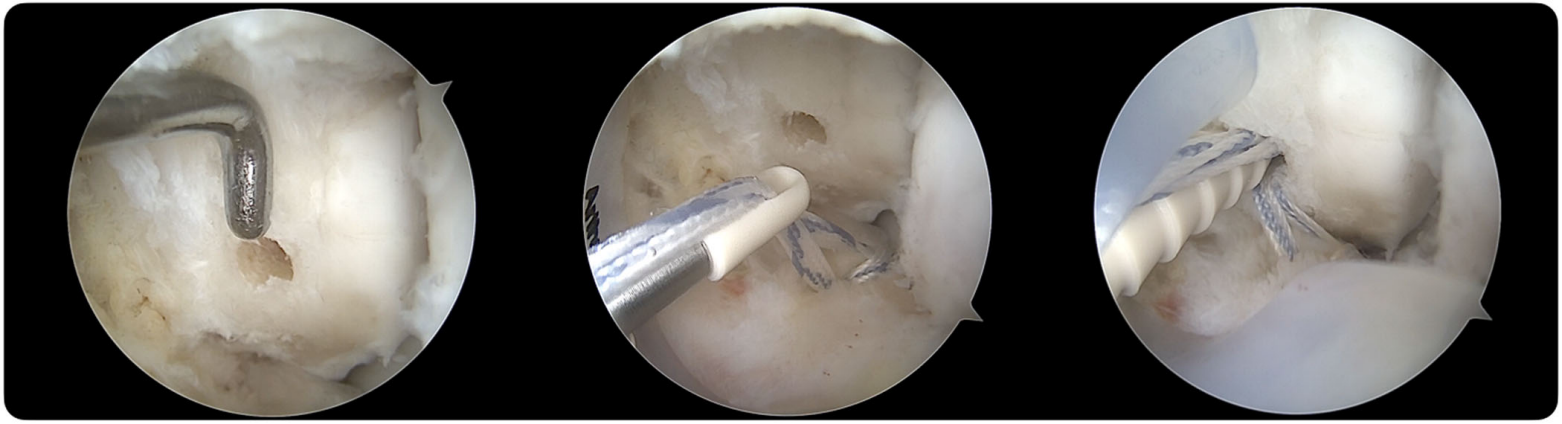
Step‐by‐step all‐inside repair of the ATFL. After drilling, the separation between the fibular physis and the anchor placement is carefully checked. Once the hole is prepared, the knotless anchor preloaded with suture is introduced by impaction. ATFL, anterior talofibular ligament.

**Figure 4:**
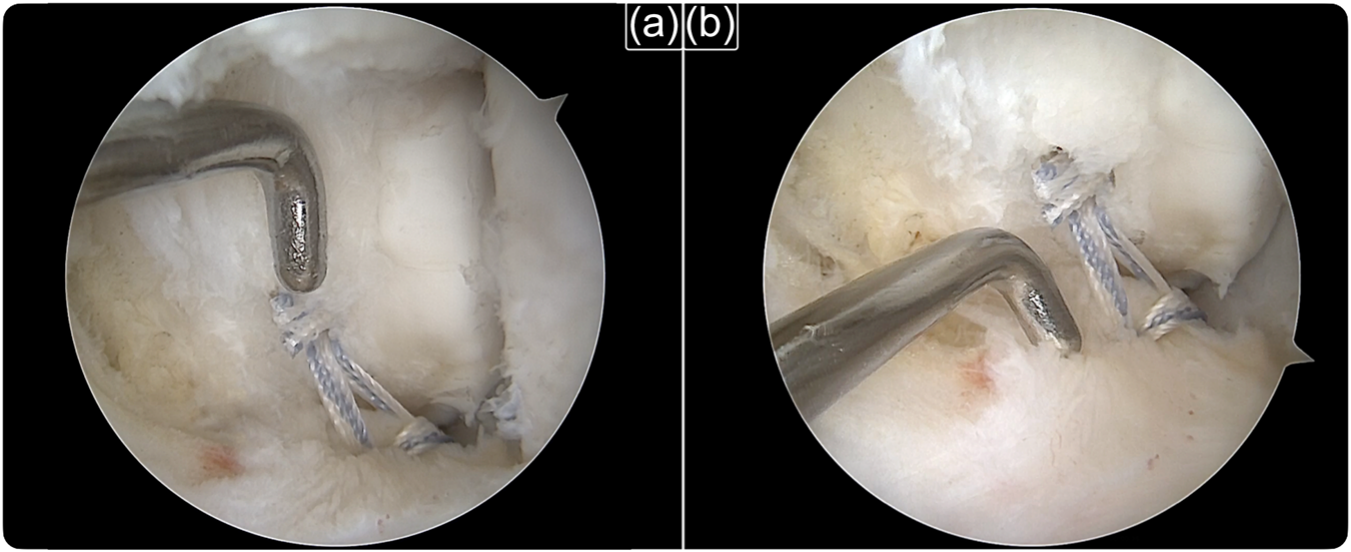
Once the repair is completed, the distance between the anchor and the physis is checked with a probe (a), and finally, the stability of the repair is tested (b).

During ligament repair, the fibular physis and the native ATFL footprint were identified. Arthroscopically, the fibular physis is typically hidden by the anterior inferior tibiofibular ligament but can be visualized just posterior to it at the level of the joint line (Figure 1). A resection of the anterior inferior tibiofibular ligament makes it possible to fully visualize the physis. To avoid damaging the growth plate, the anchor was located just distal to the fibular attachment of the anterior inferior tibiofibular ligament at a safe distance. The separation between the fibular physis and the location of the anchor was carefully checked prior to drilling. Furthermore, to prevent physeal injury, the drill was oriented parallel to the lateral gutter, plantar plane and physis with the ankle maintained at 90° dorsiflexion (Figure 2). Once the hole was drilled, the anchor preloaded with suture was introduced by impaction (Figure 3). Suture tension was adjusted prior to final anchor placement. The ATFL was reattached with the ankle held in dorsiflexion and eversion (Figure 4). In cases of concomitant CFL insufficiency, a similar technique was utilized [25]. A suture passer clamp (MiniScorpion; Arthrex) was used to grasp the CFL, and both sutures, grasping ATFL and CFL, were placed into the same anchor. ﻿Finally, sutures were cut with arthroscopic scissors, and the arthroscopic portals were closed. A removable walking boot was applied.

The walking boot was used continuously for the first 3–4 weeks, allowing partial weightbearing as tolerated with the aid of crutches starting from the immediate postoperative period. Antithrombotic prophylaxis was not administered. Formal physiotherapy commenced after boot removal at 3 weeks and included active and passive range‐of‐motion exercises, gait training, strengthening of dorsiflexion, plantarflexion, eversion and inversion, as well as balance and proprioception training. Forced inversion was avoided during the first 6 weeks. Return to sports was permitted once the full ROM, strength and neuromuscular control were restored.

### Statistical analysis

Continuous variables, including the VAS, the FFI and the FAAM‐SS, were analysed preoperatively and postoperatively. Normality of the distribution of the differences between pre‐ and postoperative values was assessed using the Shapiro–Wilk test. For normally distributed data, paired *t* tests were employed to compare pre‐ and postoperative scores. For non‐normally distributed data, the Wilcoxon signed‐rank test was used. A *p* value less than 0.05 was considered statistically significant. All analyses were performed using standard statistical software.

## RESULTS

A total of 40 patients aged 8–15 years were included in the study. Of these, 21 were female and 19 male, with a mean age of 13.8 ± 2.3 years. The right ankle was affected in 24 of the 40 cases. Patient demographics are summarized in Table 1. The mean follow‐up was 44.8 months ± 16.5 (range, 24–78), with no patients lost during the study period.

**Table 1:** Patient demographics.

No. of patients	40
Sex (male/female)	19/21
Side (right/left)	24/16
Mean age (years)	13.8 ± 2.32 (range, 8–15)

On clinical examination, a positive anterior drawer test was observed in all patients, indicating lateral ankle instability. During arthroscopy, an ATFL tear was confirmed in all cases. In addition, four patients were found to have a concomitant CFL detachment. Additional arthroscopic procedures, including synovectomy and osteophyte resection, were performed in 10 patients.

All clinical outcome scores showed statistically significant improvement (*p* < 0.001). The mean FFI improved from 35.6 ± 14.0 (range, 11–69) preoperatively to 3.9 ± 6.4 (range, 0–26) postoperatively. The mean FAAM‐SS improved from a mean of 50.3 ± 14.0 (range, 15–78) to 95.4 ± 8.3 (range, 68–100). The VAS improved from a mean of 5.4 ± 2.5 (range, 1–8) preoperatively to 0.4 ± 0.8 (range, 0–3) at final follow‐up.

The 3 months postoperative MRI showed the repaired ATFL as a linear band‐like structure running from the talar insertion to the native fibular attachment site in all cases, with no cases of physeal violation related to anchor placement.

At the final follow‐up, all patients reported subjective improvement in ankle stability. On clinical examination, the anterior drawer test was negative in all cases, and no limitations in ankle ROM were noted when compared to the contralateral side.

During the follow‐up period, no major complications requiring revision ankle surgery were observed. In addition, no lower‐limb axial deviation or clinical signs of physeal violation were detected. Minor ankle sprains occurred in eight patients and were managed conservatively with complete return to their daily and sports activities. However, no recurrence of ankle instability or persistent pain was observed in those patients. At final follow‐up, all patients were satisfied except for one patient in whom surgery did not meet expectations due to persistent pain.

## DISCUSSION

The most important contribution of this study is the demonstration that arthroscopic all‐inside ligament repair is a safe and effective treatment for ankle instability in skeletally immature patients, with minimal risk of clinically significant physeal complications. Although the presence of an open physis poses unique surgical challenges, the all‐inside technique allows for an all‐epiphyseal, anatomical repair of the ligament remnant, avoiding violation of the growth plate.

Ankle sprains and resulting chronic instability are common in skeletally immature individuals, particularly those engaged in sports [6, 17]. A recent study reported a 20% prevalence of CAI among adolescent athletes, significantly impacting ankle function [6]. This data should alarm surgeons since mechanical instability can lead to progressive joint damage, including synovitis, deltoid ligament injuries and osteochondral defects [13, 19] that may be difficult to solve and can result in reduced health‐related quality of life, cessation of physical activity and early osteoarthritis [13, 14, 15].

Initial management of ankle instability in young patients typically involves conservative treatment with aggressive rehabilitation to strengthen peroneal musculature and proprioception. In cases of failure of conservative treatment, surgical treatment is recommended. The decision to proceed with surgical stabilization should be considered not only in the presence of persistent symptoms and functional limitations but also based on the patient's age and mechanical ankle stability, with the aim of preventing further joint damage.

Surgical management in the presence of an open epiphyseal plate requires special considerations. Traditional techniques must be adapted to avoid physeal injury. In the past, various techniques have been proposed for paediatric patients, including anatomic Broström‐type repairs and reconstructions using the peroneus brevis tendon or a fibular periosteal flap [9]. Although reconstructive techniques—particularly those involving periosteal flaps—have demonstrated good clinical outcomes [18], especially in paediatric patients, it is critical to restore normal anatomy as closely as possible. For this reason, the open Broström procedure and its modifications have been considered the gold standard for treating lateral ankle instability and are generally preferred over reconstruction in both adults and children [1, 9, 16].

In the last decade, arthroscopic treatment of ankle instability has gained popularity due to several advantages, including less invasiveness, reduced postoperative pain, faster recovery and the ability to address intra‐articular pathology simultaneously.

Several recent studies have reported excellent outcomes with arthroscopic ligament repair in adults, and the all‐inside technique has been shown to achieve superior results compared to open repair [2, 10, 11, 26]. Therefore, the arthroscopic method has been advocated to replace open surgery as the gold standard treatment for ankle instability [22]. However, most of these studies focus on adult populations, and data on arthroscopic stabilization in skeletally immature patients remain limited.

Takao et al. recently reported excellent outcomes using a modified lasso‐loop technique for arthroscopic lateral ankle stabilization in skeletally immature patients [3]. Their technique employed fluoroscopic guidance to facilitate accurate anchor placement without violating the physis, resulting in significant improvement in clinical outcomes at 12‐month follow‐up. ﻿

In the present study, the arthroscopic all‐inside technique as originally described by Vega et al. [23] yielded excellent clinical outcomes with significant improvements in functional scores at a mean follow‐up of 44.84 months ± 16.50 (range, 24–78). The majority of patients returned to sports, and all but one expressed satisfaction with the outcome.

**Figure 5:**
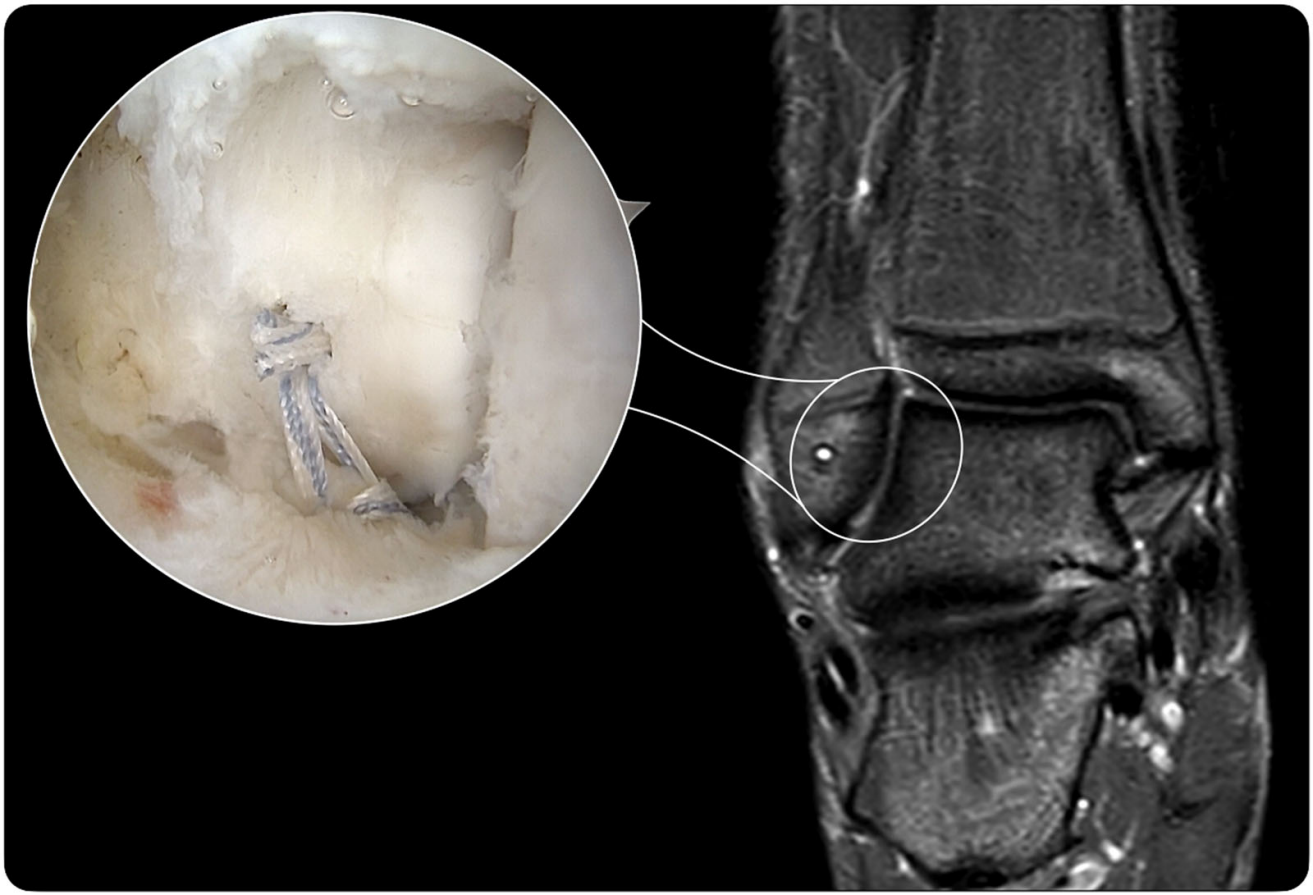
Postoperative MRI (right) and corresponding arthroscopic view (left) showing the safe distance between the fibular physis and the knotless anchor. MRI, magnetic resonance imaging

Importantly, based on clinical observations and confirmed by the current study, in most paediatric cases, the lateral ligament injury involves only the ATFL, whose anatomical insertion on the fibula is entirely epiphyseal. Therefore, it is worth emphasizing that the technique described in this study allows for an anatomical repair that naturally avoids crossing the physis, making it an ideal approach for skeletally immature patients (Figure 5). In addition, the technique as described in this study involves direct visualization of the fibular physis during arthroscopy. When drilling the tunnel for the knotless anchor, the drill is oriented from anterior to posterior, parallel to the lateral talar wall and to the plantar plane, with the ankle held at 90°. This approach ensures that the drill trajectory remains parallel to the fibular physis, avoiding growth plate injury. Notably, this can be achieved without intraoperative fluoroscopy, allowing for an all‐epiphyseal anatomical repair of the native ligament. In addition, in paediatric patients, due to the smaller size of anatomical structures, the choice of anchor size is crucial. In this study, a 2.9 × 12.5 mm anchor was used to ensure a safe distance from the physis and appropriate dimensions for the lateral malleolus.

﻿This study has several limitations. The primary limitation is that both preoperative and postoperative evaluations were conducted by a single observer, which may have resulted in an increased risk of observer‐related and measurement bias. Second, there was a wide age range among patients (8–15 years), and the lack of advanced bone age assessment (e.g., Risser or Tanner staging) may have introduced potential bias related to skeletal maturity. Third, the FFI and FAAM‐SS were originally validated in adult populations; therefore, their use in adolescents may represent a potential source of measurement bias.

Additionally, the retrospective design of the study limits the strength of the conclusions, and a larger, more homogeneous and prospectively collected cohort would certainly improve the validity of these findings. Although the results suggest physeal safety, larger cohorts are required to narrow the confidence interval around the true risk. Another limitation is the lack of postoperative weight‐bearing x‐rays, which could have helped detect subtle malalignment or growth disturbances. However, postoperative MRI was routinely obtained to assess ligament repair and to detect any physis violation.

The clinical relevance of the current study is to prove the effectiveness of arthroscopic all‐inside ligament repair in treating ankle instability in skeletally immature patients. The technique provides an all‐epiphyseal anatomical repair of the native ligament while offering the advantages of arthroscopic surgery, including treatment of concomitant intra‐articular pathology, reduced invasiveness and greater surgical precision. Therefore, arthroscopic all‐inside repair may be recommended for ankle stabilization in skeletally immature patients.

## CONCLUSIONS

This study demonstrates that ankle instability can be successfully treated using arthroscopic all‐inside ligament repair technique in skeletally immature patients. The technique provides an all‐epiphyseal anatomical repair of the ligament remnant with a low risk of physeal violation, resulting in excellent clinical outcomes.

## AUTHOR CONTRIBUTIONS

**Matteo Guelfi**: Conceptualization; methodology; writing. **Jorge Knörr**: Conceptualization; methodology. **Giovanni Ruscigno**: Writing; editing. **Francesc Malagelada**: Methodology; editing; data analysis. **Andrea Pantalone**: Conceptualization; methodology. **Jordi Vega**: Conceptualization; methodology; writing.

## CONFLICT OF INTEREST STATEMENT

The authors declare no conflict of interest.

## ETHICS STATEMENT

Ethics permission was obtained by the institutional review board of Clinica Salus, Alessandria (Italy) (IRB number 2021‐09‐020). No informed consent was needed in this study because this is a retrospective chart review of standard care procedures of de‐identified patients.

## Data Availability

The authors confirm that the data supporting the findings of the study are available within the article.

## References

[1] Bell SJ , Mologne TS , Sitler DF , Cox JS . Twenty‐six‐year results after Broström procedure for chronic lateral ankle instability. Am J Sports Med. 2006;34(6):975–978. 10.1177/0363546505282616 16399935

[2] Brown AJ , Shimozono Y , Hurley ET , Kennedy JG . Arthroscopic versus open repair of lateral ankle ligament for chronic lateral ankle instability: a meta‐analysis. Knee Surg Sports Traumatol Arthrosc. 2020;28(5):1611–1618. 10.1007/s00167-018-5100-6 30109370

[3] Chua ENL , Jujo Y , Iwashita K , Inagawa M , Lee KJ , Takao M . Ankle lateral ligament reconstruction in skeletally immature patients: technique tip. Foot Ankle Orthop. 2024;9(1):24730114241228270. 10.1177/24730114241228270 PMC10851733 38333022

[4] DiGiovanni BF , Partal G , Baumhauer JF . Acute ankle injury and chronic lateral instability in the athlete. Clin Sports Med. 2004;23(1):1–19. 10.1016/S0278-5919(03)00095-4 15062581

[5] DiGiovanni CW , Brodsky A . Current concepts: lateral ankle instability. Foot Ankle Int. 2006;27(10):854–866. 10.1177/107110070602701019 17054892

[6] Donovan L , Hetzel S , Laufenberg CR , McGuine TA . Prevalence and impact of chronic ankle instability in adolescent athletes. Orthop J Sports Med. 2020;8(2):2325967119900962. 10.1177/2325967119900962 PMC7029541 32118082

[7] Fairbanks PJ , Saito GH , Mendes AAM , Nishikawa DRC , Fonseca FCP , Prado MP . Ankle instability in pediatric and adolescent patients diagnosed with lateral malleolus avulsion fracture: analysis of clinical and functional outcomes of ligament injury repair surgery. Foot Ankle Surg. 2024;30(6):499–503. 10.1016/j.fas.2024.03.013 38632005

[8] Ferran NA , Maffulli N . Epidemiology of sprains of the lateral ankle ligament complex. Foot Ankle Clin. 2006;11(3):659–662. 10.1016/j.fcl.2006.07.002 16971255

[9] Gruskay JA , Brusalis CM , Heath MR , Fabricant PD . Pediatric and adolescent ankle instability: diagnosis and treatment options. Curr Opin Pediatr. 2019;31(1):69–78. 10.1097/MOP.0000000000000720 30531226

[10] Guelfi M , Baalbaki R , Malagelada F , Dalmau‐Pastor M , Vega J . Arthroscopic all‑inside ligament repair has similar or superior clinical outcomes compared to open repair for chronic ankle instability without concomitant intra‑articular pathology at 5 years follow‑up. Knee Surg Sports Traumatol Arthrosc. 2023;31(12):6052–6058. 10.1007/s00167-023-07621-7 37843588

[11] Guelfi M , Zamperetti M , Pantalone A , Usuelli FG , Salini V , Oliva XM . Open and arthroscopic lateral ligament repair for treatment of chronic ankle instability: a systematic review. Foot Ankle Surg. 2018;24(1):11–18. 10.1016/j.fas.2016.05.315 29413768

[12] Holland B , Needle AR , Battista RA , West ST , Christiana RW . Physical activity levels among rural adolescents with a history of ankle sprain and chronic ankle instability. PLoS One. 2019;14(4):e0216243. 10.1371/journal.pone.0216243 PMC6490934 31039184

[13] Hong CC , Tan KJ , Calder J . Chronic lateral ankle ligament instability—current evidence and recent management advances. J Clin Orthop Trauma. 2024;48:102328. 10.1016/j.jcot.2023.102328 PMC10806209 38274643

[14] Houston MN , Van Lunen BL , Hoch MC . Health‐related quality of life in individuals with chronic ankle instability. J Athl Train. 2014;49(6):758–763. 10.4085/1062-6050-49.3.54 PMC4264647 25299444

[15] Hubbard‐Turner T , Turner MJ . Physical activity levels in college students with chronic ankle instability. J Athl Train. 2015;50(7):742–747. 10.4085/1062-6050-50.3.05 PMC4532186 25898110

[16] Hunt KJ , Griffith R . Open Brostrom for lateral ligament stabilization. Curr Rev Musculoskelet Med. 2020;13(6):788–796. 10.1007/s12178-020-09679-z PMC7661567 33159666

[17] Mandarakas M , Pourkazemi F , Sman A , Burns J , Hiller CE . Systematic review of chronic ankle instability in children. J Foot Ankle Res. 2014;7(1):21. 10.1186/1757-1146-7-21 PMC3995109 24641786

[18] Mathieu P‐A , Marcheix P‐S , Vacquerie V , Dijoux P , Mabit C , Fourcade L . Reconstruction of the lateral ligaments of the ankle using a periosteal flap in children and teenagers: a midterm follow‐up survey. J Pediatr Orthop. 2015;35(5):511–515. 10.1097/BPO.0000000000000303 25171673

[19] Nishimura A , Senga Y , Fujikawa Y , Takegami N , Akeda K , Ogura T , et al. Prevalence and risk factors of ankle osteoarthritis in a population‐based study. Foot Ankle Surg. 2024;30(5):389–393. 10.1016/j.fas.2024.02.009 38453588

[20] Tanen L , Docherty CL , Van Der Pol B , Simon J , Schrader J . Prevalence of chronic ankle instability in high school and Division I athletes. Foot Ankle Spec. 2013;7(1):37–44. 10.1177/1938640013509670 24287210

[21] Vega J . Editorial commentary: arthroscopic all‐inside ligament repair is an essential technique and the emerging gold‐standard treatment for ankle instability. Arthroscopy. 2025;41:4750–4751. 10.1016/j.arthro.2025.06.026 40588177

[22] Vega J , Dalmau‐Pastor M . Editorial commentary: arthroscopic treatment of ankle instability is the emerging gold standard. Arthroscopy. 2021;37(1):280–281. 10.1016/j.arthro.2020.10.043 33384088

[23] Vega J , Golanó P , Pellegrino A , Rabat E , Peña F . All‐inside arthroscopic lateral collateral ligament repair for ankle instability with a knotless suture anchor technique. Foot Ankle Int. 2013;34(12):1701–1709. 10.1177/1071100713502322 23978706

[24] Vega J , Guelfi M , Malagelada F , Peña F , Dalmau‐Pastor M . Arthroscopic all‐Inside anterior talofibular ligament repair through a three‐portal and no‐ankle‐distraction technique. JBJS Essent Surg Tech. 2018;8(3):e25. 10.2106/JBJS.ST.18.00026 PMC6292720 30588370

[25] Vega J , Malagelada F , Dalmau‐Pastor M . Arthroscopic all‐inside ATFL and CFL repair is feasible and provides excellent results in patients with chronic ankle instability. Knee Surg Sports Traumatol Arthrosc. 2020;28(1):116–123. 10.1007/s00167-019-05676-z 31432243

[26] Zhi X , Zhang Y , Li W , Wang Y , Zou Y , Lu L , et al. Absorbable suture anchor and knotless anchor techniques produced similar outcomes in arthroscopic anterior talofibular ligament repair. Knee Surg Sports Traumatol Arthrosc. 2022;30(6):2158–2165. 10.1007/s00167-021-06855-7 35099599

[27] Zhou Y‐F , Zhang Z‐Z , Zhang H‐Z , Li W‐P , Shen H‐Y , Song B . All‐inside arthroscopic modified Broström technique to repair anterior talofibular ligament provides a similar outcome compared with open Broström‐Gould procedure. Arthroscopy. 2021;37(1):268–279. 10.1016/j.arthro.2020.08.030 32911005

